# Antimicrobial Resistance Profile of Urinary Bacterial Isolates from Hospitalized Companion Dogs Reveals a Potential Public Health Risk in South Korea

**DOI:** 10.3390/vetsci13010070

**Published:** 2026-01-10

**Authors:** Seoyoon Park, Changseok Han, Su-Man Kim, Joong-Hyun Song, Tae-Hwan Kim

**Affiliations:** 1 College of Veterinary Medicine, Chungnam National University, Daejeon 34134, Republic of Korea; seoyoon0927@naver.com (S.P.); lcid5635@naver.com (C.H.); jh.song@cnu.ac.kr (J.-H.S.); 2 Department of Biology Education, Chonnam National University, Gwangju 61186, Republic of Korea; kimsm3324@chonnam.ac.kr; 3 Institute of Veterinary Science, Chungnam National University, Daejeon 34134, Republic of Korea

**Keywords:** antimicrobial resistance, multidrug-resistant pathogens, urinary tract infection

## Abstract

Emerging antimicrobial resistance (AMR) in companion animals represents a global health concern as they serve as potential reservoirs for multidrug-resistant (MDR) bacteria, which can be transmitted to humans. Herein, we provide comprehensive surveillance data on resistance patterns in veterinary hospital settings, focusing on urinary tract infection. A total of 23 bacterial strains were isolated from urine specimens of hospitalized companion animals suspected of urinary tract infections (UTIs) between 2022 and 2024. 16S rRNA sequencing analysis revealed that *Escherichia coli* (47.8%), *Klebsiella pneumoniae* (21.7%), and *Pseudomonas aeruginosa* (8.7%) were predominant uropathogens. Minimum inhibitory concentration and minimum bactericidal concentration tests were employed to analyze AMR patterns across different classes of antibiotics. Moreover, antimicrobial susceptibility test exhibited 73.91% MDR according to the standard definition given by the Clinical and Laboratory Standards Institute (CLSI) M100 guidelines. Most Gram-negative bacteria have been shown to be resistant to beta-lactam antibiotics, especially carbapenems. Notably, an *E. coli* strain was confirmed to possess the *bla_NDM-1_* gene encoding the carbapenemase New Delhi metallo-β-lactamase. These findings support the implementation of targeted infection control measures and evidence-based treatment protocols to preserve antimicrobial efficacy in companion animal medicine to minimize potential public health risks through the One Health approach.

## Simple Summary

Antibiotic resistance in companion animals is becoming a serious public health concern. When bacteria develop resistance to multiple antibiotics, they can spread from animals to humans, making infections harder to treat. This study examined urine samples from companion animals hospitalized with urinary tract infections over two years. Three types of bacteria were most commonly found: *Escherichia coli*, *Klebsiella pneumoniae*, and *Pseudomonas aeruginosa*. Alarmingly, nearly three-quarters of these bacteria were resistant to multiple antibiotics, including some “last-resort” antibiotics called carbapenems. Additionally, one *E. coli* strain carried a dangerous resistance gene called *bla_NDM-1_*, which makes bacteria extremely difficult to treat. These findings highlight why veterinarians must use antibiotics carefully and why companion animal owners should follow treatment instructions accurately. The health of companion animals and human families is connected, as what affects one can affect the other. Better infection control in veterinary hospitals and smarter antibiotic use can help protect both animal and human health.

## 1. Introduction

Antimicrobial resistance (AMR) represents one of the most urgent global threats to both human and veterinary public health. In 2021, bacterial AMR was associated with approximately 4.71 million deaths worldwide, of which 1.14 million were directly attributable to drug-resistant infections [1]. To counter this escalating challenge, the World Health Organization (WHO) updated the Bacterial Priority Pathogens List (BPPL) in 2024 to guide research prioritization, policy development, and the discovery of novel antimicrobials. Within this framework, third-generation cephalosporin-resistant (3GCRE) and carbapenem-resistant Enterobacterales (CRE) were classified as “critical” priority pathogens due to their limited therapeutic options and significant threat to global health [2].

Growing evidence indicates that companion animals can serve as reservoirs and potential disseminators of multidrug-resistant (MDR) bacteria, posing risks of zoonotic transmission to humans. Frequently reported MDR species in companion animals include *Escherichia coli* (*E. coli*), *Staphylococcus aureus* (*S. aureus*), *Staphylococcus pseudintermedius* (*S. pseudintermedius*), *Klebsiella pneumoniae* (*K. pneumoniae*), and *Acinetobacter baumannii* [3,4,5,6,7]. Many of these belong to the ESKAPE group—pathogens renowned for their remarkable ability to evade antimicrobial action and acquire resistance through genetic mutations and mobile genetic elements, including plasmids, transposons, and integrons [8]. Such mechanisms enable the rapid spread of resistance traits across bacterial populations and host species.

Urinary tract infections (UTIs) are among the most common bacterial infections in companion animals, representing one of the leading indications for antimicrobial prescription in veterinary practice and accounting for approximately 12% of all antibiotic use in dogs [9,10]. Inappropriate or empirical antibiotic use can lead to therapeutic failure, resistance development, and increased public health risks [11]. Gram-negative bacteria dominate as the causative agents of canine and feline UTIs, with uropathogenic *E. coli* (UPEC) being the most prevalent pathogen in both uncomplicated and complicated infections. Other frequently isolated uropathogens include *K. pneumoniae*, *Proteus mirabilis* (*P. mirabilis*), *Pseudomonas aeruginosa* (*P. aeruginosa*), and *S. aureus* [12,13].

Fluoroquinolones and cephalosporins remain among the most frequently prescribed antimicrobials for UTIs; however, their extensive use accelerates the selection and dissemination of resistant strains [12,14]. Horizontal gene transfer (HGT) of extended-spectrum β-lactamase (ESBL) and carbapenemase genes plays a central role in conferring resistance to β-lactams and other antimicrobial classes [15]. Although carbapenems are rarely used in veterinary settings, carbapenemase-producing Enterobacterales have been increasingly reported in companion animals [14]. According to the Ambler classification system, β-lactamases are categorized into classes A, B, and D. The carbapenemases KPC (class A) [16,17], NDM (class B) [16,18,19], and OXA-48-like (class D) [16,20,21] β-lactamases have been detected in animal isolates, raising new challenges for treatment, infection control, and interspecies transmission.

Close interactions between humans and companion animals facilitate the bidirectional transmission of bacteria. Such transmission can occur through both direct and indirect pathways. Direct transmission involves close physical contact, such as petting, kissing, or licking, whereas indirect transmission occurs via shared household environments or veterinary settings [5,6,22]. Previous studies have demonstrated the potential for bidirectional exchange of antimicrobial-resistant bacteria between humans and companion animals [3,23], underscoring the risk of zoonotic transmission associated with close human–animal contact. Therefore, continuous monitoring of AMR in companion animals, and human surveillance, is essential for an effective One Health approach, particularly within veterinary hospitals. Despite growing global attention to AMR in veterinary contexts, much of the existing research remains geographically limited, hindering a comprehensive understanding of resistance dynamics in companion animals.

In this study, bacterial isolates were obtained from urine specimens of hospitalized companion animals to characterize AMR patterns and assess the prevalence of resistant uropathogens. Understanding these AMR profiles is crucial for guiding evidence-based therapeutic decisions, supporting antimicrobial stewardship in veterinary medicine, underscoring the potential risk for zoonotic dissemination of resistant bacteria.

## 2. Materials and Methods

### 2.1. Sample Collection and Clinical Information

A total of 51 urinary specimens were collected between 2022 and 2024 from hospitalized companion dogs and cats at Chungnam National University Teaching Hospital in Daejeon, Republic of Korea. Urine samples were collected by cystocentesis from hospitalized dogs suspected of urinary tract infection, with careful attention to prevent cross-contamination. The samples were inoculated in LB broth (#244620; BD Difco™, Franklin Lakes, NJ, USA) and then cultured aerobically at 37 °C overnight in a shaking incubator. From 51 specimens, 23 bacterial isolates were obtained and identified by 16S rRNA gene sequencing. All isolates were stored at −80 °C for future research. Each bacterial isolate was assigned a unique code consisting of the institutional abbreviation, Chungnam National University Teaching Hospital, the bacterial species name, and a sequential isolation number.

### 2.2. Identification of Bacterial Strains

Bacterial strains were identified by 16S rRNA gene sequencing conducted by Macrogen sequencing facility (Macrogen Inc., Seoul, Republic of Korea. Briefly, bacterial colonies were obtained from agar plates, and their DNA was extracted by the boiling method for analysis. PCR amplification of the 16S rRNA gene was performed using primers 27F (5′-AGAGTTTGATCMTGGCTCAG-3′) and 1492R (5′-TACGGYTACCTTGTTACGACTT-3′) [24]. Approximately 1400 base pairs of the amplified products were analyzed using the NCBI Nucleotide BLAST database (http://www.ncbi.nlm.nih.gov/BLAST/, accessed on 7 January 2026) to determine bacterial species.

### 2.3. Antimicrobial Susceptibility Test

The Kirby-Bauer disk diffusion method was used to determine the antibiotic susceptibility profile, employing Mueller-Hinton agar (MHA; MB-M1033; MBcell, Seoul, Republic of Korea). Discs of 29 different antibiotics (BD BBL™ Sensi-Disc™, BD, Franklin Lakes, NJ, USA) were used as follows: Amikacin (30 µg), Gentamicin (10 µg), Kanamycin (30 µg), Streptomycin (10 µg), Tobramycin (10 µg), Ampicillin (10 µg), Amoxicillin/Clavulanic acid (20/10 µg), Ampicillin/Sulbactam (10/10 µg), Cefazolin (30 µg), Cefaclor (30 µg), Cefoxitin (30 µg), Cefuroxime (30 µg), Cefixime (5 µg), Cefotaxime (30 µg), Ceftazidime (30 µg), Ceftriaxone (30 µg), Cefepime (30 µg); Ertapenem (10 µg), Imipenem (10 µg), Meropenem (10 µg), Aztreonam (30 µg), Chloramphenicol (30 µg), Ciprofloxacin (5 µg), Nalidixic acid (30 µg), Colistin (10 µg), Doxycycline (30 µg), Tetracycline (30 µg), Tigecycline (15 µg), Azithromycin (15 µg). Clinical isolates from animals were first inoculated in nutrient broth (NB; #234000; BD Difco™) and incubated overnight aerobically at 37 °C in a shaking incubator. The bacterial suspension was adjusted to a 0.5 McFarland standard, then diluted in 11 mL of Mueller-Hinton broth (MHB; MB-M1034; MBcell, Yongin-si, Republic of Korea) to reach approximately 1.0 × 10^5^ CFU/mL. The appropriate volume of inoculum was swabbed evenly onto the surface of MHA plates, following the CLSI guidelines [25]. Antibiotic discs were placed on the inoculated plates, which were then incubated at 37 °C for 18 h. Zones of inhibition were measured with a ruler or calipers, and susceptibility interpretations (resistant, intermediate, or susceptible) were made according to CLSI M100 breakpoint criteria. Isolates resistant to at least one agent in three or more antibiotic classes were classified as multidrug-resistant (MDR), based on criteria proposed by GBD 2021 Antimicrobial Resistance Collaborators [1].

### 2.4. Minimum Inhibitory Concentration (MIC) and Minimum Bactericidal Concentration (MBC)

The minimum inhibitory concentration (MIC) was determined using Sensititre™ Companion Animal Vet AST Plates (COMPGN1F; COMPGP1F; Thermo Fisher Scientific, Waltham, MA, USA) according to the manufacturer’s instructions. Briefly, single colonies grown on agar were suspended in phosphate-buffered saline (PBS), and the turbidity was adjusted to a 0.5 McFarland standard. This suspension was further diluted in 11 mL of MHB to achieve an approximate final concentration of 1.0 × 10^5^ CFU/mL. Antimicrobial susceptibility testing was performed against 19 antibiotics using a standardized dilution range as specified by the test plate. Plates were sealed with adhesive film and incubated at 37 °C for 18 h. To determine the minimum bactericidal concentration (MBC), 40 μL of broth from each well corresponding to the MIC value and the next higher concentration was inoculated onto MHA plates and incubated at 37 °C for 18 h. The MBC was defined as the lowest concentration at which no visible bacterial growth was observed.

### 2.5. Polymerase Chain Reaction

Isolates exhibiting carbapenem resistance were subjected to PCR to detect the presence of the *bla_KPC_*, *bla_NDM-1_*, *bla_IMP_*, and *bla_VIM_* genes. Individual colonies from each plate were suspended in 100 μL of distilled water and boiled at 100 °C for 10 min to obtain each template DNA. PCR amplification was conducted using AccuPower™ PCR PreMix (K-2016; Bioneer, Daejeon, Republic of Korea). The 20 μL reaction mixture contained 10 ng of template DNA, 2 μL of forward primer (20 μM), 2 μL of reverse primer (20 μM), and 14 μL of RNase-free water. The thermal cycling conditions consisted of an initial denaturation at 95 °C for 5 min; followed by 35 cycles of denaturation at 95 °C for 45 s, annealing at 60 °C for 45 s, and extension at 72 °C for 1 min; with a final extension at 72 °C for 10 min. PCR products were stained using RedSafe™ Nucleic Acid Staining Solution (#21141; iNtRON Biotechnology, Sungnam, Republic of Korea) and visualized on a 1.5% agarose gel under UV light. The specific primers of targeted genes are listed in Table S1 [26,27,28].

### 2.6. Statistical Analysis

There is no statistical analysis of data in this manuscript. But, the illustration was performed using GraphPad Prism version 8 (GraphPad Software, San Diego, CA, USA).

## 3. Results

### 3.1. Isolation of Bacterial Strains in Urine Specimens

Among 51 urine samples collected from dogs and cats, 21 Gram-negative and 2 Gram-positive bacterial strains were isolated exclusively from dogs (Table 1). The species with the highest prevalence were *E. coli* (47.8%) and *K. pneumoniae* (21.7%), both of which are Gram-negative bacteria. Moreover, *P. aeruginosa*, *P. mirabilis*, *Shigella flexneri* (*S. flexneri*), and *Enterobacter hormaechei* (*E. hormaechei*), which are also Gram-negative bacteria, were also isolated. In contrast, *S. pseudintermedius*, the Gram-positive bacterium, was isolated from only two specimens (8.6%).

### 3.2. Characterization of the Antibiotic Resistance Profile of the Gram-Negative Strains

Among the Gram-negative bacterial isolates, the highest rates of antibiotic resistance were observed for ampicillin (92.3%) and amoxicillin/clavulanic acid (89.5%). In contrast, all Gram-negative isolates were susceptible to amikacin (100%), while high susceptibility was also observed for gentamicin (90.5%), imipenem (95.2%), and colistin (90.0%) (Table S2).

*E. coli* isolates exhibited higher resistance to ceftazidime and ceftriaxone, whereas *K. pneumoniae* showed complete resistance (100%) to ampicillin/sulbactam, amoxicillin/clavulanic acid, and doxycycline (Table 2). Notably, all Gram-negative isolates remained susceptible to colistin (90.0%).

Additionally, several Gram-negative strains exhibited resistance to carbapenem antibiotics, including ertapenem (10.5%), imipenem (4.8%), and meropenem (19.0%). The emergence of carbapenem-resistant enterobacterales (CRE) is of particular concern, as these bacteria can rapidly disseminate carbapenemase genes that confer broad resistance. Conversely, the two *P. aeruginosa* isolates demonstrated similar resistance patterns to most Gram-negative antibiotics, except for azithromycin. Antibiotics for which intrinsic resistance is well established were excluded from the analysis of resistance rate for the AMR disc diffusion and MIC results.

**Figure 1:**
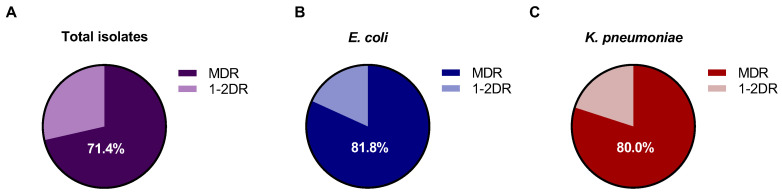
Proportion of MDR among Gram-negative isolates, *E. coli*; *K. pneumoniae*; *P. aeruginosa*; *P. mirabilis*; *S. flexneri*; *and E. hormaechei*. (**A**) Gram-negative isolates (*n* = 21); (**B**) *E. coli* isolates (*n* = 11); (**C**) *K. pneumoniae* isolates (*n* = 5).

According to the definitions proposed, 71.43% of the 21 Gram-negative bacterial isolates were classified as MDR, with the remainder being 1–2 drug resistance (1-2DR) (Figure 1A). Notably, 81.81% of the 11 *E. coli* strains (Figure 1B) and 80% of the 5 *K. pneumoniae* strains (Figure 1C) were MDR. Most of the strains presented an MDR profile and did not show an extensive drug-resistance (XDR) profile.

### 3.3. Carbapenemase Gene-Harboring Isolates

**Figure 2:**
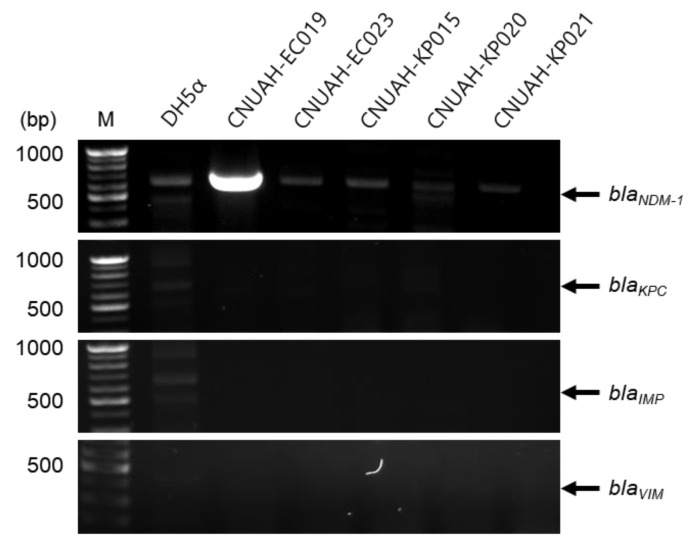
Detection and plasmid confirmation of the *bla_NDM-1_* gene in carbapenem-resistant isolates by PCR. DNA templates from each isolate were amplified using specific primers by PCR. DH5α was used as a negative control. The expected amplicon sizes and primer sequences for each target gene are provided in Table S1.

To investigate the presence of specific resistance genes, five clinical isolates initially identified as carbapenem-resistant by AMR disc diffusion testing were further analyzed using PCR (Figure 2). Unexpectedly, most isolates did not harbor known carbapenemase genes, including *bla_NDM-1_*, *bla_KPC_*, *bla_IMP_*, and *bla_VIM_*. However, isolate EC019 carried the *bla_NDM-1_* gene and exhibited strong resistance to all three carbapenem antibiotics, ertapenem, imipenem, and meropenem (Figure 2 and Figures S1–S4). Although the *bla_NDM-1_* gene is well known to be horizontally transmitted between germs via plasmids, recent studies have reported that it can also exist on the chromosome [29,30]. Since genomic DNA was extracted using a boiling method, it was not possible to determine whether *bla_NDM-1_* gene is located on a plasmid or chromosome. Further experiments are needed to determine which of the plasmids or chromosomes harbors this gene.

In addition, to investigate the strain type and potential virulence of EC019, strains with high sequence similarity to EC019 based on 16S rRNA sequencing were analyzed. The results revealed that EC019 shares 99.72% sequence identity with previously reported Enterohemorrhagic *E. coli* O157:H7 (GenBank accession: CP038319.1) or Shiga toxin-producing *E. coli* (GenBank accession: CP091018.1) according to NCBI BLAST analysis [31,32]. These findings speculate that EC019 is a highly virulent pathogen to humans and animals. However, further studies using WGS are required for in-depth analysis.

### 3.4. MIC and MBC of All Bacterial Strains

MIC or MBC of antibiotics against Gram-negative and Gram-positive isolates was presented in Table 3 or Table S3  and Table 4 or Table S4, respectively. Consistent with the AMR profiles obtained from the disc diffusion test, many isolates exhibited resistance to multiple classes of antibiotics. Notably, a high prevalence of resistance was observed against β-lactam antibiotics, including β-lactam/β-lactamase inhibitor combinations and cephalosporins. Although fluoroquinolones were not included in the AMR disc test, MIC analysis revealed relatively high levels of resistance, with fewer than half of the isolates remaining susceptible. These findings collectively indicate that most isolates displayed broad-spectrum AMR.

Among the isolates, *S. pseudintermedius* strains were identified to show resistance to oxacillin. Specifically, isolate SI027 exhibited resistance in both the AMR disc diffusion and MIC tests based on the CLSI M100 breakpoint criteria. In contrast, isolate SI017 was resistant only in the disc diffusion test but not in the MIC test. Cefoxitin is typically used as a surrogate marker for detecting oxacillin resistance in *Staphylococcus* spp., and isolates demonstrating resistance to either cefoxitin or oxacillin are categorized as methicillin (oxacillin)-resistant. However, both isolates were susceptible to penicillinase-labile penicillins, such as ampicillin, suggesting that they may behave similarly to methicillin-susceptible staphylococci, which are generally susceptible to most β-lactam antibiotics, β-lactam/β-lactamase inhibitor combinations, and cephalosporins. Given these inconsistent results, further molecular analysis, including PCR detection of *mecA* and *mecC* genes, are warranted to confirm methicillin resistance.

## 4. Discussion

This study demonstrated that bacterial isolates obtained from companion animals with urinary tract infections exhibited diverse AMR profiles, with a substantial proportion meeting the criteria for MDR. These findings highlight not only the growing concern of MDR bacteria circulating among companion animals, which have been increasingly recognized as potential reservoirs, but also the importance of accurate diagnosis, continuous surveillance, and the implementation of effective control and prevention strategies, as essential factors to help mitigate the spread of AMR in both veterinary and public health sectors.

Such AMR poses serious challenges to both veterinary and human medicine, as pathogens exhibiting MDR are often associated with increased morbidity, prolonged hospitalization, treatment failure, and even mortality. The occurrence of MDR bacteria in animals was associated with the abuse of antibiotic prescriptions and antibiotic treatment [33,34,35]. Recently, there have been few antibiotic options for referral cases from primary animal hospitals. Indeed, AMR analysis exhibited MDR of more than 70% in all isolates, more than 80% in *E. coli* or *K. pneumoniae*.

Since intrinsic resistance is a species-specific characteristic shared by all or nearly all isolates and is independent of prior antimicrobial exposure (e.g., *K. pneumoniae* is intrinsically resistant to ampicillin) [36,37], antimicrobial agents to which a species is intrinsically resistant are considered clinically ineffective, even when in vitro susceptibility is observed. *E. hormaechei* demonstrates intrinsic resistance to multiple antibiotic classes through constitutive expression of chromosomal AmpC β-lactamase, conferring natural resistance to ampicillin, amoxicillin, and first-generation cephalosporins as determined by MIC analysis [38,39]. Similarly, consistent with our results, *P. mirabilis* exhibits substantial antibiotic resistance to approximately 50% of clinical isolates, complicating clinical therapeutic options [40]. Therefore, susceptible results for intrinsically resistant organisms should be interpreted with caution when considering prescription, as they may reflect errors in organism identification or susceptibility testing rather than true antimicrobial activity [36]. Accordingly, in this study, antibiotics for which intrinsic resistance is well established were excluded from the resistance rate analysis to avoid misinterpretation of antimicrobial susceptibility results.

The WHO List of Medically Important Antimicrobials classifies carbapenems as highest-priority critically important antimicrobials (HPCIAs) and authorizes their use exclusively in human medicine [41]. A carbapenem resistance gene was detected in a urine sample from a dog. Its presence in a companion animal isolate is unlikely to be driven by direct antimicrobial selective pressure in veterinary settings. Nevertheless, the use of antibiotics approved for human medicine is common in companion animal clinical practice. Broad-spectrum antibiotics and critically important antimicrobials (CIAs) account for more than 70% of veterinary antimicrobial prescriptions [6]. Previous studies evaluating antimicrobial use in South Korea reported the 15 most frequently prescribed antibiotics across 100 companion animal clinics. Among these, cefalexin (19.75%), amoxicillin–clavulanate (18.08%), metronidazole (11.72%), amoxicillin (6.74%), cefazolin (2.61%), ampicillin (2.31%), and clindamycin (1.60%) were commonly prescribed to both humans and companion animals [42]. This practice may facilitate the development of antimicrobial resistance and interspecies transmission.

Although some carbapenem resistance genes were detected in resistant isolates, further research is needed to identify other types of carbapenem resistance genes and genes conferring resistance to the other classes of antibiotics. *bla_NDM-1_* is usually harbored in plasmids; therefore, we attempted to determine whether *bla_NDM-1_* was located on the chromosome or plasmids. However, we were unable to separate plasmid DNA from chromosomal DNA during plasmid extraction, and thus the precise genetic location of *bla_NDM-1_* could not be conclusively determined in this study. Currently, no veterinary-specific imipenem resistance breakpoints are available for companion-animal-derived isolates. Therefore, antimicrobial susceptibility was interpreted based on human clinical breakpoints, which may lead to discrepancies between disk-diffusion-based AMR profiles and MIC results. Importantly, the presence of the *bla_NDM-1_* gene does not necessarily correlate with phenotypic carbapenem resistance. Previous study reported the existence of “silent” *bla_NDM-1_*-harboring *K. pneumoniae* isolates that remain susceptible to carbapenems (e.g., imipenem, meropenem) [43]. Silent antimicrobial resistance genes refer to resistance genes harbored by bacteria that do not confer phenotypic resistance under standard susceptibility testing conditions. Consistent with this concept, previous studies have reported that certain pathogens may harbor antimicrobial resistance genes while remaining phenotypically susceptible to the corresponding antibiotics. Such discrepancies can arise from various factors, including silencing of antibiotic resistance by mutation (SARM), promoter region effect, presence of integrons, regulatory factors, and epigenetic changes [44]. Future work should focus on clarifying whether these mechanisms underlie the observed genotype–phenotype discrepancies and on precisely determining the genetic locations of antimicrobial resistance genes, which would allow a more comprehensive and detailed characterization of resistance determinants and their roles in antimicrobial resistance. 

This study appears somewhat limited in that it analyzed only samples obtained from a single tertiary veterinary hospital. Furthermore, considering that only about 50 urine samples were collected from animals with suspected UTIs over a two-year period, resulting in 23 strains, the results of the AMR profiling analysis are not generalizable. Nevertheless, the isolated strains and overall proportions are quite similar to previous reports conducted in clinical settings. *E. coli* was shown to be the predominant species that accounted for nearly half of all bacterial isolates, emphasizing its clinical significance as a major uropathogen in companion animals [3,45,46,47]. Moreover, despite the small sample size, the results demonstrate that AMR is a very critical crisis. Further studies involving larger sample sizes, diverse animal populations, and multicenter collaboration are warranted to obtain a more comprehensive understanding of AMR in companion animals. Monitoring antibiotic resistance profiles and the rational use of antibiotics in companion animals are essential for collecting data that aids in treating animal diseases. Furthermore, systematic testing of bacteria and periodic reporting of drug resistance patterns in companion animals continue to be needed for public health.

High prevalence of AMR, particularly multidrug resistance and ESBL production, among hospitalized companion animal uropathogens poses significant therapeutic challenges and potential zoonotic risks. The increasing trends in resistance to critically important antimicrobials underscore the urgent need for enhanced antimicrobial stewardship programs, diagnostic-guided therapy, and comprehensive surveillance systems in veterinary healthcare. These findings support the implementation of targeted infection control measures and evidence-based treatment protocols to preserve antimicrobial efficacy for the treatment of UTIs in companion animal medicine while minimizing public health risks through a One Health approach.

## Figures and Tables

**Table 1:** Pathogenic bacterial isolates and the corresponding origins of their isolation are summarized.

Strains	Species	Specimen	Breeds	Age	Sex
CNUAH-EC002	*Escherichia coli*	Urine	Maltese	16 y	SF
CNUAH-EC003	*Escherichia coli*	Urine	Jindo	9 y	SF
CNUAH-EC004	*Escherichia coli*	Urine	Mixed	9 y	SF
CNUAH-EC005	*Escherichia coli*	Urine	Labrador retriever	11 y	SF
CNUAH-EC019	*Escherichia coli*	Urine	Jindo	12 y	SF
CNUAH-EC023	*Escherichia coli*	Urine	Mixed	7 y	SF
CNUAH-EC025	*Escherichia coli*	Urine	Yorkshire terrier	12 y	SF
CNUAH-EC026	*Escherichia coli*	Urine	Shiba Inu	6 y	SF
CNUAH-EC029	*Escherichia coli*	Urine	Welsh Corgi	8 y	CM
CNUAH-EC030	*Escherichia coli*	Urine	Spitz	7 y	CM
CNUAH-EC031	*Escherichia coli*	Urine	Maltese	15 y	SF
CNUAH-KP014	*Klebsiella pneumoniae*	Urine	Mixed	12 y	M
CNUAH-KP015	*Klebsiella pneumoniae*	Urine	Mixed	12 y	M
CNUAH-KP020	*Klebsiella pneumoniae*	Urine	Mixed	5 y	SF
CNUAH-KP021	*Klebsiella pneumoniae*	Urine	Mixed	14 y	SF
CNUAH-KP022	*Klebsiella pneumoniae*	Urine	Mixed	14 y	SF
CNUAH-PA009	*Pseudomonas aeruginosa*	Urine	Mixed	13 y	SF
CNUAH-PA010	*Pseudomonas aeruginosa*	Urine	Mixed	13 y	CM
CNUAH-PM028	*Proteus mirabilis*	Urine	Maltese	13 y	SF
CNUAH-SF024	*Shigella flexneri*	Urine	Beagle	7 y	SF
CNUAH-EH032	*Enterobacter hormaechei*	Urine	Yorkshire terrier	8 y	M
CNUAH-SI017	*Staphylococcus pseudintermedius*	Urine	Jindo	12 y	SF
CNUAH-SI027	*Staphylococcus pseudintermedius*	Urine	Maltese	13 y	SF

CM, Castrated male; SF, spayed female; M, Male.

**Table 2:** Antibiotic resistance profiles for each isolated species.

Antibiotics	Resistance (%)			
	Total	*E. coli*	*K. pneumoniae*	*P. aeruginosa*
	(*n* = 21)	(*n* = 11)	(*n* = 5)	(*n* = 2)
Amikacin	0.0	0	0	0
Gentamicin	9.5	18.2	0	0
Kanamycin	23.8	27.3	0	100
Streptomycin	47.6	36.4	60	100
Tobramycin	14.3	9.1	0	0
Ampicillin *	92.3	90.9	IR	IR
Amoxicillin/Clavulanic acid *	89.5	90.9	100	IR
Ampicillin/Sulbactam	90.5	81.8	100	100
Cefazolin *	72.2	81.8	60	IR
Cefaclor	76.2	81.8	60	100
Cefoxitin *	60.0	36.4	60	100
Cefuroxime	71.4	63.6	40	100
Cefixime	76.2	81.8	60	100
Cefotaxime *	47.4	63.6	20	IR
Ceftazidime	38.1	36.4	0	0
Ceftriaxone *	47.4	54.5	0	IR
Cefepime	14.3	18.2	0	0
Ertapenem *	10.5	18.2	0	IR
Imipenem	4.8	9.1	0	0
Meropenem	23.8	9.1	60	0
Aztreonam	42.9	9.1	0	0
Chloramphenicol *	36.8	9.1	60	IR
Ciprofloxacin	52.4	45.5	60	0
Colistin *	10.0	0	0	0
Doxycycline	61.9	45.5	100	100
Tetracycline *	61.1	36.4	100	IR
Tigecycline *	44.4	0	0	IR
Nalidixic Acid	81.0	45.5	60	100
Azithromycin	57.1	18.2	0	50

* Antibiotics for which the isolates with intrinsic resistance. These were excluded from the analysis of resistance rate. IR: intrinsic resistance.

**Table 3:** MIC of antibiotics against Gram-negative isolates.

Antibiotics	AMI	AUG2	FAZ	POD	TAZ	LEX	CHL	DOX	ENRO	GEN	IMI	MAR	ORB	P/T4	PRA	TET	SXT
	MIC (μg/mL)																
EC002	<4	>8/4	>32	>8	>16	<0.25	4	1	0.25	1	<1	0.25	<1	<8/4	<0.25	<4	>4/76
EC003	<4	>8/4	>32	>8	>16	<0.25	<2	1	<0.12	1	<1	0.25	<1	<8/4	<0.25	<4	>4/76
EC004	<4	8/4	>32	>8	<4	>16	4	>8	>4	>8	<1	>4	>8	<8/4	>2	>16	>4/76
EC005	<4	8/4	>32	>8	<4	2	<2	8	>4	>8	<1	>4	>8	<8/4	2	>16	>4/76
EC019	8	>8/4	32	>8	>16	>16	8	>8	>4	>8	<1	>4	>8	>64/4	>2	>16	>4/76
EC023	<4	>8/4	>32	>8	16	<0.25	4	1	<0.12	2	<1	0.25	<1	<8/4	>2	<4	>4/76
EC025	<4	2/1	2	<1	<4	<0.25	4	0.5	<0.12	1	<1	<0.12	<1	<8/4	<0.25	<4	>4/76
EC026	<4	>8/4	>32	>8	<4	>16	<2	0.5	0.25	4	<1	0.5	2	<8/4	<0.25	<4	>4/76
EC029	<4	>8/4	>32	>8	16	>16	32	8	>4	1	<1	>4	>8	16/4	>2	>16	>4/76
EC030	<4	>8/4	>32	>8	>16	>16	4	1	0.25	1	<1	0.25	<1	<8/4	>2	<4	>4/76
EC031	<4	2/1	2	<1	<4	8	4	1	<0.12	1	<1	<0.12	<1	<8/4	<0.25	<4	>4/76
KP014	<4	4/2	4	>8	<4	8	4	>8	<0.12	0.5	<1	<0.12	<1	<8/4	<0.25	>16	>4/76
KP015	<4	4/2	4	<1	<4	8	4	>8	<0.12	0.5	<1	<0.25	<1	<8/4	<0.25	>16	>4/76
KP020	<4	>8/4	>32	>8	<4	>16	>32	>8	<0.12	0.5	<1	4	>8	<8/4	2	>16	>4/76
KP021	<4	>8/4	>32	>8	<4	>16	>32	8	>4	0.5	<1	4	>8	<8/4	2	>16	>4/76
KP022	<4	>8/4	>32	>8	<4	>16	>32	8	>4	0.5	<1	4	>8	16/4	2	>16	>4/76
PA009	<4	>8/4	>32	>8	<4	<0.25	16	1	0.25	<0.25	<1	<0.25	<1	<8/4	<0.25	<4	2/38
PA010	<4	>8/4	>8	>8	<4	>16	32	8	0.25	<0.25	<1	<0.12	<1	<8/4	<0.25	<4	>4/76
PM028	<4	1/0.5	2	>8	>16	8	32	>8	>4	4	<1	>4	>8	<8/4	2	>16	>4/76
SF024	<4	2/1	2	<1	>16	8	<2	0.5	>4	1	<1	<0.25	>8	<8/4	2	<4	>4/76
EH032	<4	>8/4	>32	4	>16	>16	4	>8	4	0.5	<1	2	8	<8/4	1	>16	>4/76

MIC (μg/mL) of 19 antibiotics against the isolates was determined using the broth microdilution method. Background color: green, susceptible; yellow, intermediate; red, resistant; blue, intrinsic resistant. AMI, Amikacin; AUG2, Amoxicillin/clavulanic acid 2:1; FAZ, Cefazolin; POD, Cefpodoxime; TAZ, Ceftazidime; LEX, Cephalexin; CHL, Chloramphenicol; DOX, Doxycycline; ENRO, Enrofloxacin; GEN, Gentamicin; IMI, Imipenem; MAR, Marbofloxacin; ORB, Orbifloxacin; P/T4, Piperacillin/tazobactam constant 4; PRA, Pradofloxacin; TET, Tetracycline; SXT, Trimethoprim/sulfamethoxazole.

**Table 4:** MIC of antibiotics against Gram-positive isolates.

Antibiotics	SI017	SI027
	MIC (μg/mL)	
AMP	<0.25	<0.25
FAZ	<2	<2
CHL	16	16
CLI	>4	>4
DOX	>0.5	>0.5
ENRO	0.5	0.5
GEN	<4	<4
MAR	<1	<1
MIN	2	>2
NIT	<16	64
OXA+	<0.25	>2
PEN	<0.06	1
PRA	<0.25	<0.25
RIF	<1	<1
TET	>1	>1
SXT	>4/76	<2/38
VAN	<1	>16

MIC was determined for Gram-positive isolates using the broth microdilution method. Background color: green, susceptible; yellow, intermediate; red, resistant. AMP, Ampicillin; FAZ, Cefazolin; CHL, Chloramphenicol; CLI, Clindamycin; DOX, Doxycycline; ENRO, Enrofloxacin; GEN, Gentamicin; MAR, Marbofloxacin; MIN, Minocycline; NIT, Nitrofurantoin; OXA+, Oxacillin + 2%NaCl; PEN, Penicillin; PRA, Pradofloxacin; RIF, Rifampin; TET, Tetracycline; SXT, Trimethoprim/sulfamethoxazole; VAN, Vancomycin.

## Data Availability

The original contributions presented in this study are included in the article and Supplementary Materials. Further inquiries can be directed to the corresponding author.

## Supplementary Materials

The following supporting information can be downloaded at: https://www.mdpi.com/article/10.3390/vetsci13010070/s1, Figure S1: PCR image of blaNDM-1 (621 bp); Figure S2: PCR image of blaKPC (785 bp); Figure S3: PCR image of blaIMP (587 bp); Figure S4: PCR image of blaVIM (389 bp); Table S1: Primer sequences used are listed; Table S2: Antibiotic resistance profiles for total Gram-negative isolates; Table S3: MBC results for total Gram-negative isolates; Table S4: MBC results for total Gram-positive isolates.

## Abbreviations

The following abbreviations are used in this manuscript:

AMR	Antimicrobial resistance
CLSI	Clinical and laboratory standards institute
CRE	Carbapenem-resistant Enterobacterales
ESBL	Extended-spectrum beta-lactamase
HGT	Horizontal gene transfer
MBC	Minimum bactericidal concentration
MIC	Minimum inhibitory concentration
MDR	Multidrug-resistant
MHA	Mueller-Hinton agar
MHB	Mueller-Hinton broth
3GCRE	Third-generation cephalosporin-resistant
UTIs	Urinary tract infections
UPEC	Uropathogenic *E. coli*
WHO	World Health Organization
XDR	Extensive drug-resistance
