# Supplementary material for: Antimicrobial Resistance Profile of Urinary Bacterial Isolates from Hospitalized Companion Dogs Reveals a Potential Public Health Risk in South Korea
Source: Vet Sci. 2026 Jan 10;13(1):70. doi: 10.3390/vetsci13010070 (PMC12846553; doi:10.3390/vetsci13010070)
Supplement: Supplementary file 1 [file vetsci-13-00070-s001.zip › Supplementary Figures.pdf]

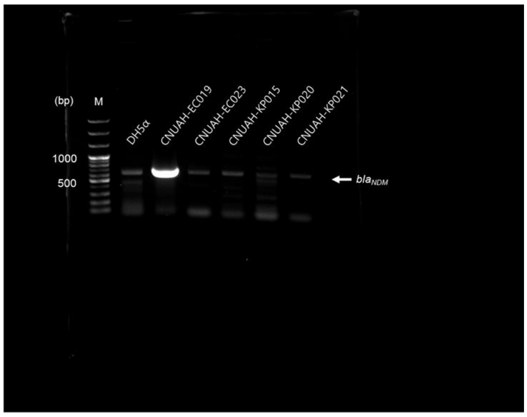

Figure S1. PCR image of *bla*$_{NDM-1}$ (621 bp)

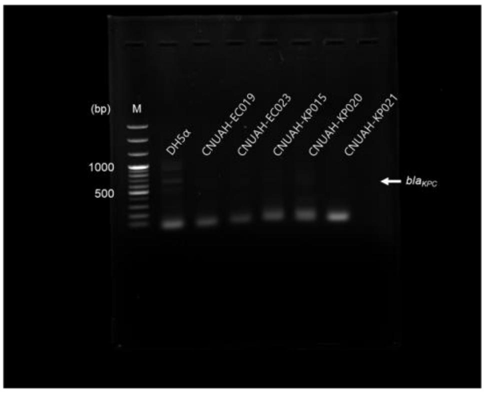

Figure S2. PCR image of *bla*$_{KPC}$ (785 bp)

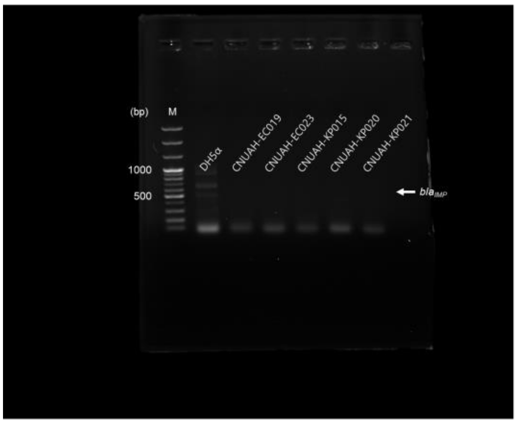

Figure S3. PCR image of of *bla*$_{IMP}$ (587 bp)

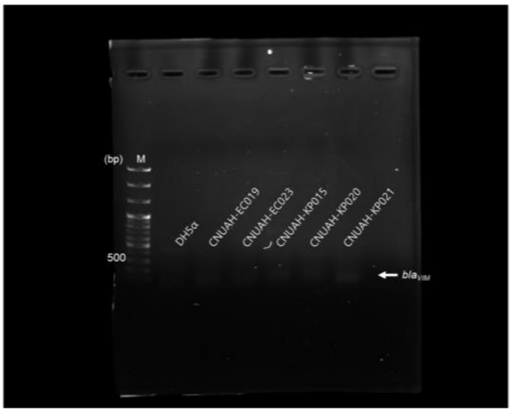

Figure S4. PCR image of bla_{VIM} (389 bp)
